# Long-term survival of a patient with gastric cancer with bone marrow metastasis receiving S-1 plus oxaliplatin beyond three years: a case report and literature review

**DOI:** 10.3389/fonc.2024.1449212

**Published:** 2024-08-06

**Authors:** Hirotaka Suto, Yumiko Inui, Atsuo Okamura

**Affiliations:** ^1^ Department of Medical Oncology, Hyogo Cancer Center, Hyogo, Japan; ^2^ Department of Medical Oncology/Hematology, Kakogawa Central City Hospital, Hyogo, Japan

**Keywords:** gastric cancer, bone marrow metastasis, S-1 plus oxaliplatin, micrometastasis, survival

## Abstract

**Background:**

Bone marrow metastasis (BMM) of gastric cancer (GC), which is the most common cause of disseminated intravascular coagulation (DIC) among solid tumors, has a poor prognosis. Studies on prognostic improvement beyond one year in patients with GC with BMM are limited. This is the first report of a patient who survived over three years after 30 months of S-1 plus oxaliplatin (SOX) therapy for GC with BMM.

**Case Report:**

The patient was a 72-year-old woman who presented with anemia and high levels of alkaline phosphatase (ALP) and carbohydrate antigen 19-9 (CA19-9). Detailed examination led to the diagnosis with BMM of GC uncomplicated by DIC and the SOX regimen was initiated in November 2018. After six cycles, she was switched to S-1 monotherapy, and both ALP and CA19-9 levels reached normal by November 2019. However, computed tomography in April 2021 showed multiple bone metastases. Therefore, she was switched to paclitaxel-based therapy. In November 2021, the patient was further switched to nivolumab monotherapy, but she succumbed due to DIC in March 2022.

**Conclusion:**

GCs with BMM are prone to DIC, and the SOX regimen, which includes S-1 with efficacy against micrometastases, may constitute a safe and effective treatment modality.

## 1 Introduction

Gastric cancer (GC) remains a major health concern, being the fifth most common cancer worldwide, and the fourth leading cause of cancer-related mortality (1). Bone marrow metastasis (BMM) of GC is considered a rare entity (2, 3); however, it is the most common cause of disseminated intravascular coagulation (DIC) among solid tumors and has a poor prognosis (4–6). DIC is a clinical manifestation of various underlying diseases pathologically activating the coagulation system. DIC is characterized by multiple thrombi in microvessels, microcirculatory failure leading to organ damage, and wasting of coagulation factors and platelets causing bleeding tendency (7). The clinical course is rapid, and the prognosis for untreated patients is reported to be within one month (3). Previously, fluorouracil/leucovorin (5-FU/LV) regimens and 5-FU + methotrexate regimens have been reported for patients with BMM of GC, albeit with little efficacy and yielding poor prognosis ranging from 2 to 6 months (7–11). The advent of cisplatin and oxaliplatin has improved the overall prognosis of GC (12, 13); however, there are limited studies on improvement in the prognosis of patients with BMM with GC beyond 1 year (14, 15).

The present report demonstrated the case of a patient who survived over three years after 30 months of S-1 plus oxaliplatin (SOX) therapy for BMM of GC.

## 2 Case report

The patient was a 72-year-old woman who underwent total gastrectomy and splenectomy for GC in February 2012. The histopathological diagnosis was tubular 2, moderately differentiated cancer (Union for International Cancer Control classification, 7th edition) (16), and ERB-B2 receptor tyrosine kinase 2-negative, tubular, moderately differentiated cancer (the World Health Organization classification of 2019) (17). The pathological staging indicated pT2N0M0, stage IB (Union for International Cancer Control classification, 7th edition). She did not experience any recurrence for a while. However, she visited the department in October 2018 due to worsening anemia and increased serum alkaline phosphatase (ALP) levels. A computed tomography (CT) scan revealed no identifiable causes of anemia. However, bone marrow biopsy revealed the presence of solute carrier family 4 member 1/member 3-positive and caudal type homeobox 2-positive cancer cells, prompting the diagnosis of BMM of GC given her history of GC surgery. She had not been tested for *Helicobacter pylori* infection and had no history of cancer except GC. She also had no family history of suspected hereditary tumors. The authors have previously reported on her diagnostic course (18).

At the initial visit, she did not present with DIC. Her fecal occult blood was negative, and she had progressive normocytic anemia, serum ALP elevation, and abnormally high carbohydrate antigen 19-9 (CA19-9) levels. The results of hematological tests were as follows: Hemoglobin =8.5 g/dL, mean corpuscular volume =93.0 fL, platelet count =24.3x10^4^/µL, prothrombin time-International Normalized Ratio =1.01, fibrinogen =303 mg/dL, fibrin/fibrinogen degradation products =20.9 µg/mL, D-dimer =24.4 µg/mL, serum ALP =4,197 IU/L and serum CA19-9 = 303.8 IU/mL (**Table 1**). Physical examination revealed no bilateral leg edema suspicious of deep vein thrombosis, nor were there signs of dyspnea or chest pain suspicious of pulmonary embolism.

**Table 1:** Laboratory data at the time of her visit to our department.

Blood components	Patient		Normal range
Complete blood count			
White blood cell	4760	/μL	3300–8600
Red blood cell	270 x 10^4^	/μL	386–492 x10^4^
Hemoglobin	8.5	g/dL	11.6–14.8
Hematocrit	25.1	%	35.1–44.4
Mean corpuscular volume	93.0	fL	83.6–98.2
Platelet	24.3 x 10^4^	/μL	15.8–34.8 x10^4^
Neutrophil	49.0	%	40.0–70.0
Lymphocyte	41.0	%	20.0–50.0
Monocyte	7.0	%	0.0–10.0
Eosinophil	3.0	%	1.0–5.0
Basophil	0.0	%	0.0–1.0
Coagulation test			
Activated partial thromboplastin time	26.0	s	26.0–38.0
Prothrombin time	101.6	%	70.0–130.0
Fibrinogen	303	mg/dL	200–400
Fibrin/fibrinogen degradation products	20.9	μg/mL	0.0–5.0
D-dimer	24.4	μg/mL	0.0–1.0
Biochemistry			
Total protein	7.1	g/dL	6.6–8.1
Albumin	4.1	g/dL	4.1–5.1
C-reactive protein	0.02	mg/dL	0.00–0.14
Aspartate transaminase	23	IU/L	13–30
Alanine transaminase	12	IU/L	7–23
Alkaline phosphatase	4197	IU/L	106–322
Total bilirubin	0.4	mg/dL	0.4–1.5
Lactate dehydrogenase	269	IU/L	124–222
Blood urea nitrogen	23.2	mg/dL	8.0–20.0
Creatinine	0.60	mg/dL	0.46–0.79
Na (sodium)	139	mEq/L	138–145
K (potassium)	4.6	mEq/L	3.6–4.8
Cl (chlorine)	103	mEq/L	101–108
Creatine kinase	128	IU/L	41–153
Amylase	124	IU/L	44–132
Glucose	99	mg/dL	73–109
Carcinoembryonic antigen	3.3	ng/mL	0.0–5.0
Carbohydrate antigen 19-9	303.8	IU/mL	0.0–37.0

**Figure 1:**
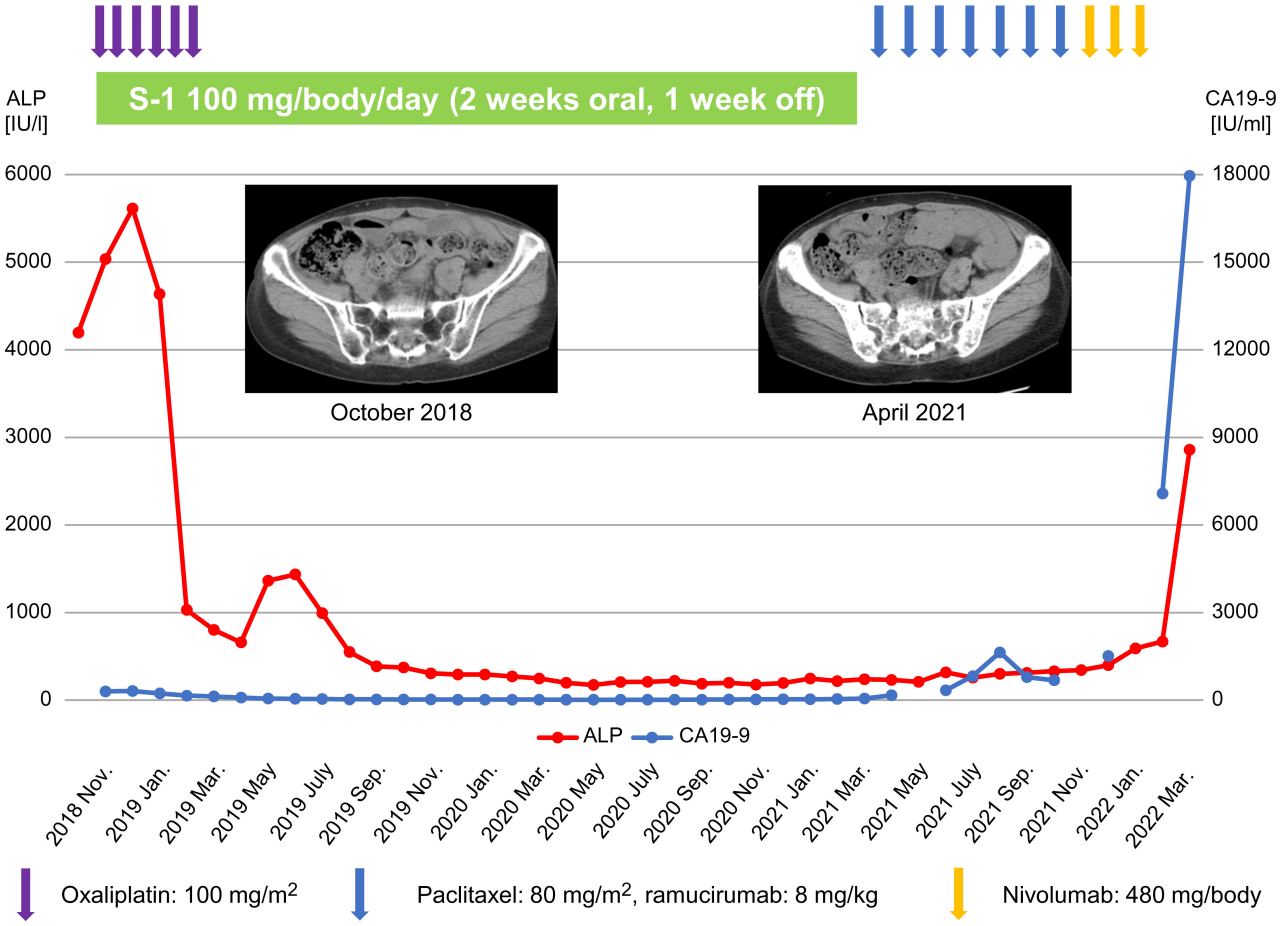
Treatment course of the patient and changes in ALP and CA19-9 levels. ALP, alkaline phosphatase; CA19-9, carbohydrate antigen 19-9.

Treatment with SOX regimen was initiated for BMM of GC in November 2018. The SOX regimen consisted of S-1 100 mg/body, weight/day, orally on days 1-14 and oxaliplatin 100 mg/m^2^ orally on day 1, for one cycle of 21 days. After 3 cycles of the SOX regimen, the ALP and CA19-9 levels decreased to 1031 IU/L and 159.4 IU/mL, respectively. Bone marrow examination performed in January 2019 failed to detect any cancer cells. However, oxaliplatin was discontinued after 6 cycles and S-1 monotherapy was administered because the peripheral neuropathy worsened to grade 3 on the Common Terminology Criteria for Adverse Events version 5.0 (19). At the time of oxaliplatin discontinuation, the ALP and CA19-9 levels were 802 IU/L and 130.1 IU/mL, respectively. However, blood tests performed in May 2019, for instance, two months after the change to S-1 monotherapy, revealed ALP and CA19-9 levels of 1,363 IU/L and 55.3 IU/mL, respectively. CT performed in the same month demonstrated no obvious recurrent lesions. Thereafter, both ALP and CA19-9 levels declined, and blood tests performed in December 2019 showed an ALP level of 293 IU/L and a CA19-9 level of 18.7 IU/mL. Bone marrow examination conducted in the same month also revealed no cancer cells. Follow-up CT imaging was performed every 3-6 months but did not find evidence of any apparent recurrent lesions. However, in April 2021, blood tests showed CA19-9 elevation to 167.5 IU/mL and CT depicted multiple bone metastases. Therefore, considering S-1 failure, the treatment was changed to weekly paclitaxel (wPTX) + ramucirumab (RAM). The wPTX + RAM regimen consisted of wPTX 80 mg/m^2^ on days 1, 8, and 15, and RAM 8 mg/kg on days 1 and 15 of a 28-day cycle. The CA19-9 levels decreased after a transient increase, however, in November 2021, they elevated to 1,516.6 IU/mL and the ALP increased to 400 IU/L. The wPTX + RAM regimen was then switched to nivolumab monotherapy in the same month; however, in January 2022, the CA19-9 level increased to 7,081.3 IU/mL and the ALP level increased to 670 IU/L. Therefore, nivolumab monotherapy was discontinued and replaced with best supportive care (BSC) alone. In the following month (February 2022), the patient’s CA19-9 and ALP levels surged to 17,961.1 IU/mL and 2,860 IU/L, respectively. She succumbed to DIC in March 2022 (**Figure 1**). Written informed consent was obtained from the patient’s family for the publication of the present case report.

## 3 Discussion

The case of a patient who survived for over 3 years after 30 months of SOX therapy for GC with BMM was presented.

GC with BMM is a rare manifestation that has not been investigated by any prospective studies; therefore, it lacks an established treatment (2, 3). Complications of BMM in solid tumors include anemia, thrombocytopenia, and DIC. While blood transfusions have been reported to be effective for managing anemia and thrombocytopenia and recombinant thrombomodulin for treating DIC, the main principle of DIC management is the appropriate treatment of the causative disease (20, 21). Despite no strict definition of GC bone marrow micrometastasis (22), we diagnosed this case as GC bone marrow micrometastasis because no obvious lesion was found on CT imaging, and recurrent GC was diagnosed only through bone marrow examination, as reported by Macadam et al. (23). Although this case was not complicated by DIC, the absence of a thrombus on CT and the presence of not only elevated D-dimer levels but also elevated fibrin/fibrinogen degradation products and ALP suggested that the patient was pre-disposed to DIC. Retrospective studies and case series of five or more cases, published in English, have reported that the prognosis of BSC alone in patients with GC complicated by BMM is poor, with an overall survival (OS) of 1 to 2 months. The prognosis of patients with GC with concomitant DIC is even worse, with an OS of ~1 month without treatment. The median OS for patients treated with 5-FU-based, taxane-based, and platinum-based regimens tends to be 2 to 6 months, longer than that of patients treated with BSC alone (**Table 2**). Conversely, SOX therapy, which was administered to this patient, is a widely used treatment in Asia, as the G-SOX and SOPP trials have demonstrated non-inferiority of efficacy to S-1 plus cisplatin (SP) combination therapy (32, 33). The G-SOX trial reported that the toxicity was grade 3 or greater anemia in 15.1% of the SOX group and 32.5% of the SP group, and grade 3 or higher thrombocytopenia in 10.1% of the SOX group and 10.4% of the SP group, and was well tolerated, even in older patients (32, 34). Thus, the SOX regimen is relatively easier to administer than SP therapy, even in the presence of anemia or DIC. S-1 used in this regimen is reportedly more effective than 5-FU against undifferentiated cells prone to BMM in GC (35). In this case, treatment was initiated before the onset of DIC, and because S-1 was administered for a long period of time, the same treatment could be continued for 30 months, and the patient survived for over 3 years.

**Table 2:** Studies or case series on gastric cancer with bone marrow metastasis.

Author	Regimen	N	Median age (range), years	DIC (+)	MST (range)
Yeh et al. (11)	5-FU-based	5	62 (30–65)	5	6 (2–13) months
Hironaka et al. (24)	5-FU+MTX	9	52 (34–66)*	9	113 days
Chao et al. (25)	EEPFL	6	40 (36–71)	6	28 (12–32) weeks
Tokar et al. (8)	5-FU-based	6	49 (32–56)	6	14.5 (1–32) weeks
Kim et al. (3)	Various chemotherapy	9	47 (26–68)*	8 (39)	67 (5–252) days
	BSC alone	9			20 (2–137) days
Huang et al. (26)	5-FU based	19	53 (31–72)	19	3 (0.5–17) months
Rhee et al. (27)	Platinum-based	14	45 (29–66)	14	99 days
	BSC alone	7	68 (24)–72	7	16 days
Takashima et al. (7)	5-FU+MTX	22	56 (26–75)	22	154 (126–180) days
Kwon et al. (28)	Various chemotherapy	16	46 (24–61)*	NA	121 (3–383) days
	BSC alone	10		NA	11 (2–34) days
Hwang et al. (29)	5-FU or Taxane-based	19	51 (25–68)	19	61 (50–72) days
	BSC alone	49	58 (26–78)	49	9 (6–16) days
Ekinci et al. (14)	CF	1	22	NA	20 days
	DCF	1	45	NA	53 days
	BSC alone	3	47 (42–55)	NA	47 days (34–61)
Sugiyama et al. (30)	SP	2	58 (50–66)	2	152.5 (114–191) days
	FOLFOX	3	38 (35–66)	3	257 (131–313) days
Zhai et al. (31)	Various chemotherapy	21	NA	21	NA
	BSC alone	15	NA	15	2.067 months
Takahashi et al. (15)	FOLFOX	5	67 (39–71)	5	115 (83–324) days

5-FU, fluorouracil; BSC, best supportive care; CF, cisplatin plus fluorouracil; DCF, docetaxel plus cisplatin plus fluorouracil; DIC, disseminated intravascular coagulation; EEPFL, etoposide plus epirubicin plus cisplatin plus fluorouracil plus leucovorin; FOLFOX, fluorouracil plus leucovorin plus oxaliplatin; MST, median survival time; MTX, methotrexate; NA, not available; SP, S-1 plus cisplatin

*Median age of all patients in the study.

Recently, immune checkpoint inhibitors (ICIs) have been introduced for the treatment of GC, which reportedly improve prognosis (36, 37). The Japanese government approved nivolumab as a first-line treatment for GC in November 2021. Therefore, ICIs could not be used as a first-line treatment for this patient. Based on the results of the KEYNOTE-061 trial (38), a paclitaxel-based therapy was preferred for second-line treatment, and therefore, nivolumab was used as the third-line treatment in this patient. Because cancer cells regulate the immune environment, ICIs may be effective against GC with BMM (39). Huang et al. (40) reported two cases of GC with BMM treated with ICIs with a cytotoxic anticancer drug, however, the effect of ICI therapy alone on BMM remains unknown. Although a combined positive score is currently used as an indicator of the effectiveness of ICIs on GC, it is not a perfect biomarker (41). Sato et al. investigated biomarkers in the microenvironment of durable responders of ICIs in GC and reported that the effect tends to be sustained in cases with a high T cell/regulatory T cell (Treg) ratio. ICIs may be effective in cases with a high T cell/Treg ratio in BMM of GC (42). Moreover, established effective regimens for GC with BMM are lacking and the results of a phase II trial exploring the efficacy of 5-FU plus docetaxel therapy, which is currently underway, are awaited (43). As a limitation, case reports, including this study, lack external validity. Therefore, S-1-based chemotherapy may not be able to provide long-term control of bone marrow dissemination in gastric cancers such as the present case. It is necessary to examine whether there is an overlap between the molecular pathology of gastric cancer prone to bone marrow dissemination and that of gastric cancer for which S-1 is effective.

In conclusion, patients with GC with BMM are prone to anemia and DIC and may be better managed with a SOX regimen. Furthermore, S-1 monotherapy is effective against micrometastases, and therefore, chemotherapy containing S-1 may prolong the survival of patients with GC with BMM.

## Data availability statement

The original contributions presented in the study are included in the article. Further inquiries can be directed to the corresponding author.

## Ethics statement

The studies involving humans were approved by The Institutional Review Board Kakogawa Central City Hospital Ethics Committee. The studies were conducted in accordance with the local legislation and institutional requirements. The patient’s family provided written informed consent to participate in this study. Written informed consent was obtained from the individual(s) for the publication of any potentially identifiable images or data included in this article.

## Author contributions

HS: Conceptualization, Investigation, Methodology, Writing – original draft, Writing – review & editing. YI: Data curation, Investigation, Writing – review & editing. AO: Conceptualization, Data curation, Investigation, Methodology, Writing – review & editing.
